# Playing Edcraft at Home: Gamified Online Learning for Recycling Intention during Lockdown

**DOI:** 10.12688/f1000research.72761.1

**Published:** 2021-09-06

**Authors:** Kin Meng Cheng, Ah Choo Koo, Junita Shariza Mohd Nasir, Shen Yuong Wong

**Affiliations:** 1 Faculty of Creative Multimedia, Multimedia University, Cyberjaya, Selangor, Selangor, 63100, Malaysia; 2 Department of Electrical and Electronics Engineering, Xiamen University Malaysia, Sepang, Selangor, 43900, Malaysia

**Keywords:** Recycling intention, Gamified learning, Youth, Digital natives, Qualitative, Focus group discussion, Edcraft, Inter-rater reliability

## Abstract

**Background:** Recycling is a way to sustain ecosystems. There are substantial studies on recycling intention due to the continual growth of unethical and unsustainable waste disposal. Creative approaches to recycling awareness activities should be made to fulfil youths’ increasing interest in and demand for recycling. The main objective of this study is to explore the factors related to youths’ recycling intentions after experiencing a gamified online recycling learning activity, Edcraft Gamified Learning (EGL). Gamified recycling education is believed to be a practical and engaging approach for youths.

**Methods:** 100 students participated in EGL, consisting of two levels of plastic crafting and recycling activities. They experienced online EGL at home between May and September in 2020, during the COVID-19 pandemic total lockdown in Malaysia, namely, Movement Control Order (MCO). 29 participants were selected to participate in five focus group discussions (FGDs) with five to eight participants per session to explore their opinions towards gamified learning, motivation and recycling intention.

**Results:** This paper reports the findings of the FGDs. A codebook was developed based on the codes from the FGD feedback. The codes were rated by two raters, followed by an assessment of inter-rater reliability and thematic analysis. The findings emerged and were confirmed with four themes as factors that influence recycling intention. They are gameful experience, social influence, intrinsic motivation, and extrinsic motivation.

**Conclusion: **The dependent variable, recycling intention, was connected to the four themes to verify the conceptual framework. One limitation of the study was the design of the EGL activity, which was only carried out over two days with two levels of gamified recycling education, as students had concurrent academic online classes during that period.

## 1 Introduction

Most countries have long ago adopted recycling to preserve the environment’s health. ^1^ Recycling behaviour has promoted a positive attitude towards the environment within society. Around the world, municipal waste production correlates with a country’s economic growth. Statistics show that pro-environmental behaviour, awareness, and intentions are the most direct measures for coping with daily household waste production. Nonetheless, plastic packaging increased 53% internationally during the lockdown in the pandemic situation between March and June 2020. ^2^

A study conducted in 2017 and 2018 shows a negative attitude towards recycling in many countries, claiming that recycling education and infrastructure are insufficient. ^3, 4^ However, the negative attitude had caused severe destruction to the environment, as they are often more reactive and ‘always forgot’ or ‘do not care’ about the act of recycling when there is no support from recycling infrastructure, facilities or programmes. ^5^

This research aims to examine the factors that influence youths’ recycling intention after experiencing a gamified online learning activity, Edcraft Gamified Learning (EGL), and to explore the effectiveness of gamified learning about recycling within a research framework. ^6^

## 2 Literature review

### 2.1 Youths as agents of change

Youths between the ages of 15 and 24 comprise 17% of the world’s population, and they are the critical agents of change for the future, especially on climate change. ^7^ Youth has been understood as the responsible group to bring the benefits of society in the world, taking its place in attitudes, behaviours, knowledge and skills. ^6^ Greta Thunberg, a young climate and environmental activist, continues to lead, promote and motivate young people’s awareness and behavioural changes towards pro-environmental actions. She encourages her followers to use digital platforms for pro-environmental awareness and actions. ^8, 9^ Thunberg’s actions have also influenced some Malaysian youths to take climate action locally, such as that taken by Klima Action Malaysia (KAMY). ^10^ The youth-led green and climate movements have created a new revolution for positive environmental change, as evidenced by their influence and leadership on social media.

**2.1.1 Youths as digital activists for positive change**

Youth populations are exposed to vast amounts of information, and multimedia and social media are recognised by these “digital natives”. ^11^ The long hours youth spent online surfing the internet, socialising and playing video games in the virtual world during lockdowns due to the pandemic has increased tremendously. The long period spent by the youths in the virtual world has created an effective platform to influence their peers through video games and social media.

Studies have shown that youths opt to spend time playing video games online to avoid adverse psychological effects while confined at home during the pandemic. ^12, 13^ Young people should be exposed to positive activities to create an impact for any form of good cause, including pro-environmental initiatives. The initiatives can be done online, as demonstrated by youth leaders such as Thunberg and KAMY.

### 2.2 Recycling and gamified learning

Waste separation and recycling are methods to curb problems associated with environmental hazards. Recycling is also a way to save economic and environmental resources. Hence, the importance of recycling behaviour has been studied, and it has been found that one of the most critical factors for achieving the effectiveness of recycling programmes is public participation, ^3^ which was studied by this research as people’s intention to participate in recycling. Recycling is seen as problematic by some, who may perceive it as time-consuming and something that requires considerable effort. ^14, 15^

Germany and Sweden are among the countries that were positively impacted by their recycling programme in educating and engaging the people in waste separation and reached 90% of active participants in waste separation activity. ^16, 17^ Gamified learning is considered to engage people in recycling, and Table 1, below, shows four different gamified environmental education programmes.

**Table 1.** Review of studies of gamified learning research in the recycling area.

Gamified learning recycling research	Findings
Make waste fun again! A gamification approach to recycling ^18^	Feedback, awards, achievements, collaborative and competitive features from video games are all important in closing the gap between waste management behaviour and knowledge.
How to encourage recycling behaviour? The case of WasteApp: a gamified mobile application ^19^	Adopting a gamified sustainability app directly and significantly impacts the intention depending on the expected social benefits and perceived risks.
English for Specific Purpose: Revitalising Climate Change Awareness Using Digital Literacy and Gamification for Engineering Faculty in Papua ^20^	Digital literacy and gamification can be used as a policy tool and developed into a learning model to raise climate change awareness.
Incentives for Plastic Recycling: How to Engage Citizens in Active Collection. Empirical Evidence from Spain ^21^	Results show people can be influenced and their recycling habits changed through varied, effective, and innovative incentive schemes.

From the findings in Table 1, the common traits of gamified environmental awareness learning are that gamification can potentially influence the adoption of a positive recycling intention.

*Recycling intention*

Some people perceive recycling as a time-consuming activity. This study will make use of gamification to engage the youth involvement in recycling, and change their “always forgot” and “do not care” attitude towards recycling intention. ^5^

### 2.3 Conceptual framework

**Figure 1.**
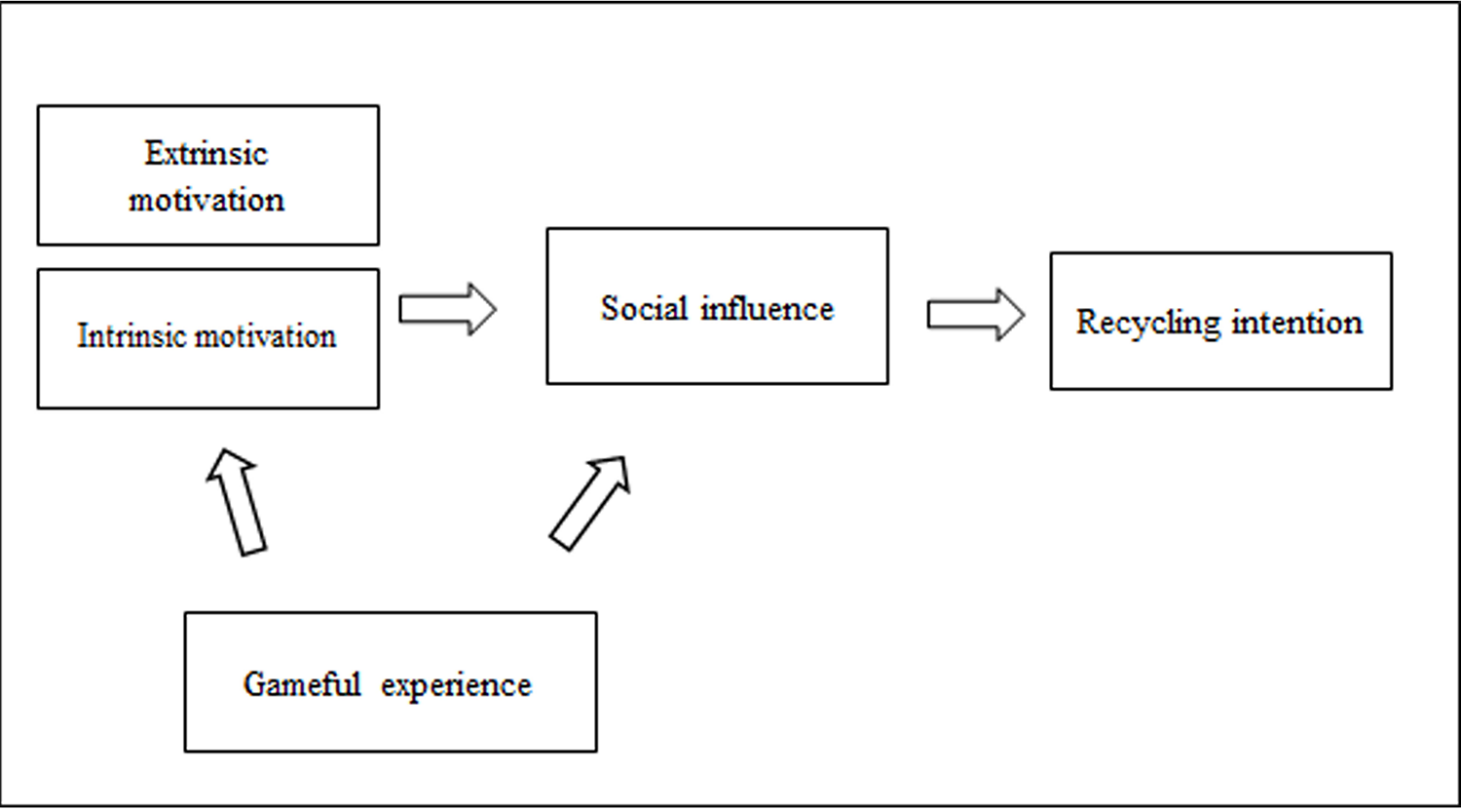
The conceptual framework showing four key themes or concepts related to recycling intention.

In Figure 1, the study has conceptualised a framework of recycling intention initiated by gamified learning. It is conceptualised that the youths’ motivation is from family and friends, and that gamification plays a part in avoiding the negative psychological effects that may occur during times when people’s movement is restricted while also helping manage the bulk of household waste.

There are two research questions (RQ) from the research:

1) How effective is the online gamified recycling activity towards youths’ recycling intention?
2) What are the factors of recycling intention that leads to the research conceptual framework?

## 3 Methods

The qualitative study relates to the two-level online gamified recycle crafting activity, namely Edcraft Gamified Learning (EGL) activity during the COVID-19 pandemic lockdown between May and September 2020. EGL is an activity where participants were required to undergo two days, two levels and each level one craft activity. Participants were also given a day to work on each of their craft freely and creatively.

This activity involved 100 youths from three institutions, one high school and two colleges in Selangor, Malaysia, who were 16–23 years old and staying at home due to the pandemic. Participants were recruited from their schools and collegesthrough the activity recruitment poster and “Word-of-Mouth”, with the help of the teachers and lecturers.

They provided written consent to participate on the day of the activity and were informed that the data would be for research and publication use. After experiencing two-level EGL, 29 students were purposively selected as FGD participants. Participants with excellent achievement, commitment and attention to the activity were selected for the FGD.

### 3.1 Prior to data analysis

Recordings were made of each of the five online FGD sessions. Each session lasted for 50–70 minutes, and could be listened to for a second time to identify recurring data patterns. Participants were given instructions beforehand and were informed several days before the FGD. The researcher was also given consent by each of the participants to conduct the FGD sessions. Recorded sessions were transcribed from audio to text. Later, the transcripts were coded and compiled into a codebook for inter-rater reliability assessment.

**3.1.1 Coding assessment**

*Inter-rater reliability assessment*

To obtain code consensus from the raters, the degree of agreement scores, 0 and 1, were measured by two raters. Both raters were trained to perform their task by identifying the target behaviour from the transcripts. ^22^ According to, ^23, 24^ the value of 0.78 in Table 2 shows the kappa statistics of agreement that were analysed by the IBM Statistical Package for Social Sciences Statistics (SPSS) Version 26 software ( IBM SPSS Statistics, RRID:SCR_019096). An open source alternative to SPSS is GNU PSPP. The statistics show substantial or very good agreement relative to the kappa statistics.

**Table 2.** Kappa statistics of agreement.

Symmetric measures				
	Value	Asymptotic standard error ^a^	Approximate T ^b^	Approximate significance
Measure of agreement kappa	0.786	0.206	4.895	0.000
N of valid cases	37			

^a^ Not assuming the null hypothesis.

^b^ Using the asymptotic standard error assuming the null hypothesis.

### 3.2 Thematic analysis

Once the coding assessment had been done, the 37 codes were familiarised, then further sorted into 13 categories and organised as themes based on repeated patterns and similarities in their nature. Thematic analysis was used in the qualitative analysis of the FGDs to identify, organise, describe, and report themes for more trustworthy. Themes were reviewed for the second and third time to gain insightful and accurate findings that match the research questions. ^25^

## 4 Results and discussion

Findings from the FGDs revealed four main themes, and these four themes are the independent variables or factors that influence the youths’ recycling intention. They are as follows:

1) Engagement with the gamified recycling activity,
2) Intrinsic motivation,
3) Extrinsic motivation, and
4) Social influence.

Recycling intention is the dependent variable. All responses from the four themes were to answer RQ 1, explore the effectiveness of online gamified recycling activity that leads to recycling intention and verify the conceptual framework in Figure 1. The 13 categories were related to the participants’ responses, as shown in Appendix A.

29 students from the FGDs shared their insights, responses, opinions and experiences online on how they acted towards the activity, the recycling issue in Malaysia, in FGDs during the COVID-19 pandemic.

### 4.1 Themes and categories

Each theme was listed in this section, and the categories were discussed based on the participants’ FGD responses. Insights from the interviews were highlighted to create a progressive construct on the conceptual framework to explore the ideas and meaning from the participants. All the verbatim were directly quoted from their original meaning.

**4.1.1 Engagement from gamified Learning activities**

When the researcher asked about their engagement on gamified learning activities, Participant P said it is crucial not to fall behind and feel ashamed. Therefore, they tend to work harder: “Yeah I think it is very important if not I will also would like you know so like I actually put more effort.” Participant R, too, was engaged because of their friend’s ranking and they valued the interaction between peers: “Your friend has a higher ranking and … work a little bit harder to get a higher rank...” Participant T stated that “We use game to compare...then it will have missions to work on, then it has the leaderboard from it, and reward, all has it in the Edcraft. I think this fulfills the standard of gameful.” Participant T had experienced video games and compared the fun of the gamified learning activity to a video game.

**4.1.2 Intrinsic motivation**

Participant K made a strong statement about the accomplishment of the EGL activity: “Yes, I feel it gives me the sense of accomplishment … I keep on doing...about 6-7 hours until I upload the video … I feel the sense of accomplishment...” Several participants felt the happiness from accomplishment more than obtaining the physical reward. Recognition has intrinsically motivated the students, and they appreciate what they have earned through hard work. “I think rewards are important … certificate they will be useful … I think you can put it in your resume.” Additionally, enjoyment is part of the participants’ emotions. They immersed themselves in the activity and felt a sense of pleasure. Participant O was triggered by the activity and enjoyed the process of making a craft out of unused recyclable items: “After participated the Edcraft … it taught me that bottle can turn into other things...”

**4.1.3 Extrinsic motivation**

Participants stayed motivated all the way to the end of the activity because they were promised a certification and prizes as rewards for completion. A reward is a form of extrinsic motivation as a temporary driver for them to recycle for participant J: “I am aiming for the certificate … the cert is very important...” Participant Q mentioned motivation from rewards: “I think after organising Edcraft, it will cultivate the awareness of recycling. Just like vase they need to create by themselves, need to spend money and expensive. If use plastic, it looks beautiful and quite practical as well.”. This response shows that the participant appreciates their crafting and building up their confidence to recycle.

**4.1.4 Social Influence**

Participants are competitive when they are trying their best to earn a reward within a challenging environment among a group of friends. They influenced each other, and EGL has shown its social influence in competition: “I think it kind of affects in a positive way for youth … you will feel like want to do better than them.” Some participants take competition, points and rankings seriously: “I have some friend, first round only got 11 points then second round same points as me 41 points … they are so serious, persist and not giving up.”

**4.1.5 Recycling intention among youth**

Participant A agreed that habit plays a role in recycling. “That is because they just do it all in habit … we do have like a monthly sort of activity in our community.” This clearly demonstrates that Participant A has done this for some time and is committed to the activity due to habit. Likewise, Participant D shows the expression of habit, saying, “every year always got talk about environment and the 3R so that’s how we all grew that habit of recycling …” There is a participant who believes that recycling is an activity that needs to be cultivated from an early age, following role models such as teachers, parents and friends. According to Participant F, “what motivates the young people to do recycling mostly comes from family. If your family practice then the children would probably just follows.” There are some participants who mentioned following teachers’ rules, being punished, recycling for the sake of rules or orders from elders: “teacher will punish the class which is not doing recycling … the person will receive punishment by sending to recycling center to wash recycling item.” Participant K loved the idea of an online EGL recycling activity: “I think after organizing Edcraft, it will cultivate the awareness of recycling … If use plastic, it looks beautiful and quite practical as well.” By having EGL, recycling awareness awakened participants’ thoughts about having fun recycling.

### 4.2 Factors influencing recycling intention

Five topics from the FGD based on the codes were finalised into four themes:

1) Gameful experience,
2) Intrinsic motivation,
3) Extrinsic motivation, and
4) Social influence.

The four main themes are the main factors that influence recycling intention to address RQ 2.

The responses show that participants were engaged with the activity. They felt the fun of the video game-like experience and were socially influenced by competition among their friends to get into the action of recycling and recycling intention.

**Figure 2.**
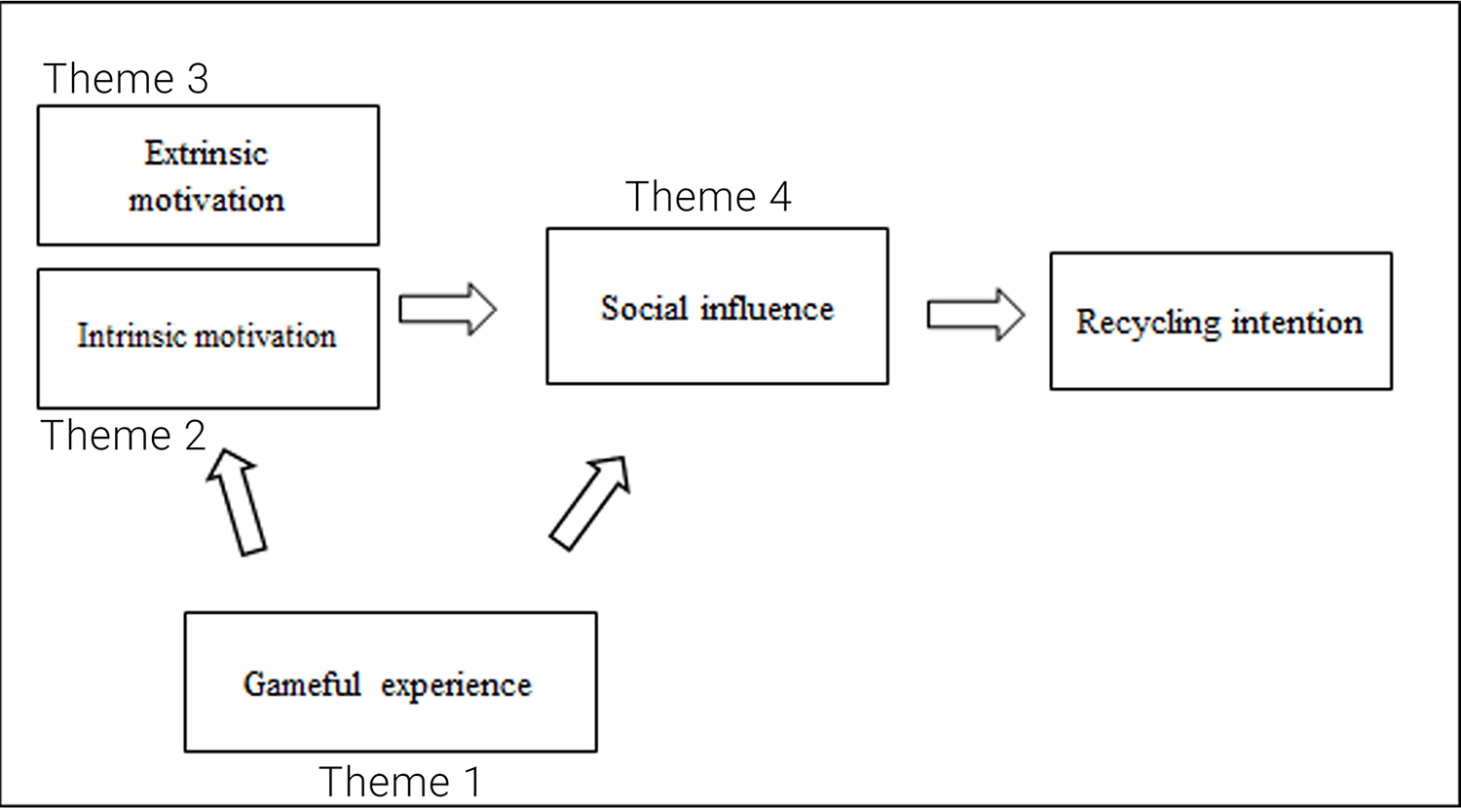
Verified conceptual framework categorised into four themes.

The collected data and themes were used to verify, enrich and create the “flow”, referring to the conceptual framework in Figure 1 and factors that influence recycling intention from RQ 1 to address RQ 2, as shown in Figure 2.

## 5 Implications and conclusion

Young people are part of social change, and they bring an impact to the betterment of the environment through recycling activities. The incorporation of gamified learning for improving recycling intention can be brought about through social influence and intrinsic and extrinsic motivations. This study serves as a starting point for researchers to dig deeper into the elements that influence intention and behavioural change among youths. The method used in this study is FGDs. This research used inter-rater reliability on the coding agreement and thematic analysis to reduce ambiguities and develop quality themes and frameworks that lead to well-grounded gamified online recycling learning. However, recycling activity requires commitment, and the two-day, two-level activity represented a short time for attitude development. It is hoped that with the incorporation of gamified online learning, the impact of social influence on pro-environmental awareness and recycling, similar to the achievements of Greta Thunberg and KAMY, can spread to more young people around the world. Cultivation of recycling behaviour requires a longer-term gamified activity to effectively close the gap between fun learning and recycling intention.

## Data availibility

### Underlying data

OpenDans: Online Gamified Learning: Focus Group Discussion Data set, May - August 2020. https://doi.org/10.17026/dans-xzu-as8s. ^26^

This project contains the following underlying data:

• Data file 1. (Cohen kappa reliability output in.spv format)
• Data file 2 to 6. (Focus Group Discussion Transcription in pdf.)
• Data file 7 to 10 (interrater reliability in.doc, .dat, .sav, and.sps format)
• Data file 11 (invitation letter for interrater)

Data are available under the terms of the Creative Commons Zero “No rights reserved” data waiver (CC0 1.0 Public domain dedication).

## Ethical approval

Ethical approval (EA) number from Multimedia University (approved by secretariat of Research Ethics Committee): EA2642021.

## References

[1] Veizer J : Sedimentation in geologic history: Recycling vs. evolution or recycling with evolution. *Contrib. to Mineral. Petrol.* 1973;38(4):261–278. 10.1007/BF00373592

[2] Filho WL : COVID-19 and waste production in households: A trend analysis. *Sci. Total Environ.* 2021; vol.777. 10.1016/j.scitotenv.2021.145997 PMC7895713 33676209

[3] Almasi A : Assessing the knowledge, attitude and practice of the kermanshahi women towards reducing, recycling and reusing of municipal solid waste. *Resour. Conserv. Recycl.* 2019; vol.141, no.November 2018, pp.329–338. 10.1016/j.resconrec.2018.10.017

[4] Yukalang N Clarke B Ross K : Barriers to effective municipal solid waste management in a rapidly urbanizing area in Thailand. *Int. J. Environ. Res. Public Health* .2017;14(9):9–14. 10.3390/ijerph14091013 28869572 PMC5615550

[5] Kumar D Kumar SA : Why Don’t People Recycle-A Comparative Study Between The United States of America and India. *Int. J. Soc. Sci. Econ. Res.* 2018; (2):6692–6712. Reference Source

[6] Ho E Clarke A Dougherty I : Youth-led social change: Topics, engagement types, organizational types, strategies, and impacts. *Futures* .2015;67:52–62. 10.1016/j.futures.2015.01.006

[7] Fernandez G Thi TTM Shaw R : Climate Change Education: Recent Trends and Future Prospects. In: *Education for Sustainable Development and Disaster Risk Reduction.* Shaw R Oikawa Y , Eds. Tokyo: Springer Japan;2014, pp.53–74.

[8] Finnegan W : Environmental activism goes digital in lockdown – but could it change the movement for good?. *The Conversation.* 2020. (accessed 1st June, 2021). Reference Source

[9] Greta Thunberg: Who is she and what does she want?. *BBC.* 2020. (accessed 1st June, 2021). Reference Source

[10] Ragunathan Y : Inspiring Stories from Malaysian Youth Leaders. 2020. (accessed 4th June, 2021). Reference Source

[11] Yang S Lee JW Kim HJ : Can an online educational game contribute to developing information literate citizens?. *Comput. Educ.* 2021; vol.161, no.October 2020, p.104057. 10.1016/j.compedu.2020.104057

[12] Wong HY : Relationships between severity of internet gaming disorder, severity of problematic social media use, sleep quality and psychological distress. *Int. J. Environ. Res. Public Health.* 2020;17(6):1–13. 10.3390/ijerph17061879 32183188 PMC7143464

[13] Brooks SK : The psychological impact of quarantine and how to reduce it: rapid review of the evidence. *Lancet.* 2020;395(10227):912–920. 10.1016/S0140-6736(20)30460-8 32112714 PMC7158942

[14] Wang Z Guo D Wang X : Determinants of residents’ e-waste recycling behaviour intentions: Evidence from China. *J. Clean. Prod.* 2016; vol.137, no.February 2009, pp.850–860. 10.1016/j.jclepro.2016.07.155

[15] Strydom WF : Barriers to household waste recycling: Empirical evidence from South Africa. *Recycling.* 2018; vol.3, no.3. 10.3390/recycling3030041

[16] Stoeva K Alriksson S : Influence of recycling programmes on waste separation behaviour. *Waste Manag.* 2017;68:732–741. 10.1016/j.wasman.2017.06.005 28619237

[17] Schwarz-Herion O Practical Action Nepal : A case study on successful municipal solid waste management in industrialized countries by the example of karlsruhe city, germany. *J Eng. Ann.* 2008; no.year, pp.1–59. Reference Source

[18] Miralem Helmefalk JR : Make Waste Fun Again! A Gamification Approach to Recycling. *Conf. Int. Conf. ArtsIT, Interactivity Game Creat. Int. Conf. Des. Learn. Innov.* 2020; vol.328 LNICST, no.July, pp.785–787. 10.1007/978-3-030-53294-9

[19] Lidia A : How to Encourage Recycling Behaviour? The Case of WasteApp: A Gamified Mobile Application. 2018; pp.1–20. 10.3390/su10051544

[20] Betaubun M Nasrawati N : English for Specific Purpose: Revitalizing Climate Change Awareness Using Digital Literacy and Gamification for Engineering Faculty in Papua. *J. Iqra’ Kaji. Ilmu Pendidik.* 2020; vol.5, no.2, pp.68–82. 10.25217/ji.v5i2.1181

[21] Gibovic D Bikfalvi A : Incentives for Plastic Recycling: How to Engage Citizens in Active Collection. Empirical Evidence from Spain. *Recycling.* 2021;6(2):29. 10.3390/recycling6020029

[22] Popping R : Introduction to Interrater Agreement for Nominal Data. 2019. 10.1007/978-3-030-11671-2

[23] Landis JR Koch GG : The Measurement of Observer Agreement for Categorical Data. *Biometrics.* Jan. 1977;33(1):159–174. 10.2307/2529310 843571

[24] Regier DA : DSM-5 field trials in the United States and Canada, part II: Test-retest reliability of selected categorical diagnoses. *Am. J. Psychiatry.* 2013;170(1):59–70. 10.1176/appi.ajp.2012.12070999 23111466

[25] Braun V Clarke V : Using thematic analysis in psychology. *Qual. Res. Psychol.* Jan. 2006;3(2):77–101. 10.1191/1478088706qp063oa

[26] Cheng KM : C (Multimedia University) (2020): Online Gamified Learning: Focus Group Discussion Data set, May - August 2020. *DANS.* 10.17026/dans-xzu-as8s

