# Supplementary material for: Playing Edcraft at Home: Gamified Online Learning for Recycling Intention during Lockdown
Source: F1000Res. 2021 Dec 20;10:890. Originally published 2021 Sep 6. [Version 2] doi: 10.12688/f1000research.72761.2 (PMC8729030; doi:10.12688/f1000research.72761.2)
Supplement: Peer review history

# Peer review: Reviewer response for version 2

**DOI:** 10.5256/f1000research.78749.r100362

**Published:** 2021-12-21

**Authors:** Ariffin Abdul Mutalib

**Affiliations:** 1 Multimedia, Innovation, and Digital Humanities Research Unit, School of Multimedia Technology and Innovation, Universiti Utara Malaysia, Sintok, Malaysia

The improvement is accepted for indexing.

Is the work clearly and accurately presented and does it cite the current literature?

Partly

If applicable, is the statistical analysis and its interpretation appropriate?

Not applicable

Are all the source data underlying the results available to ensure full reproducibility?

No

Is the study design appropriate and is the work technically sound?

Partly

Are the conclusions drawn adequately supported by the results?

Partly

Are sufficient details of methods and analysis provided to allow replication by others?

Partly

Reviewer Expertise:

HCI, Usability, Multimedia in Teaching and learning

I confirm that I have read this submission and believe that I have an appropriate level of expertise to confirm that it is of an acceptable scientific standard.

# Peer review: Reviewer response for version 2

**DOI:** 10.5256/f1000research.78749.r116167

**Published:** 2021-12-20

**Authors:** Herdiyan Maulana

**Affiliations:** 1 Faculty of Psychology, Universtias Negeri Jakarta, Jakarta Selatan, Indonesia

I am happy with the authors revision. Their paper now is ready for full indexing.

Is the work clearly and accurately presented and does it cite the current literature?

Partly

If applicable, is the statistical analysis and its interpretation appropriate?

Not applicable

Are all the source data underlying the results available to ensure full reproducibility?

Yes

Is the study design appropriate and is the work technically sound?

Partly

Are the conclusions drawn adequately supported by the results?

Partly

Are sufficient details of methods and analysis provided to allow replication by others?

Partly

Reviewer Expertise:

Thematic analysis study, Well-being, Online mental health services, well-being, social psychology

I confirm that I have read this submission and believe that I have an appropriate level of expertise to confirm that it is of an acceptable scientific standard.

# Peer review: Reviewer response for version 1

**DOI:** 10.5256/f1000research.76364.r96007

**Published:** 2021-11-17

**Authors:** Ariffin Abdul Mutalib

**Affiliations:** 1 Multimedia, Innovation, and Digital Humanities Research Unit, School of Multimedia Technology and Innovation, Universiti Utara Malaysia, Sintok, Malaysia

In general, the paper sounds like an empirical study, but the process does not sound technically sufficiently. Please consider the following to improve the paper:

1. The introduction can be better addressed by discussing the domain of study first, followed with the context of the study. The aim of the study should be stated in the section as well.
2. The literature review should also focus on the domain of study, showing the gap (that the study taps into).
3. The methods are too general and lack justification. Some parts need strong justifications, like sampling.
4. The results and discussion are too brief. Perhaps, you can focus on one factor and discuss more deeply.
5. Implications should compare the findings in this paper with those previously found by others.

Is the work clearly and accurately presented and does it cite the current literature?

Partly

If applicable, is the statistical analysis and its interpretation appropriate?

Not applicable

Are all the source data underlying the results available to ensure full reproducibility?

No

Is the study design appropriate and is the work technically sound?

Partly

Are the conclusions drawn adequately supported by the results?

Partly

Are sufficient details of methods and analysis provided to allow replication by others?

Partly

Reviewer Expertise:

Human-Computer Interaction

I confirm that I have read this submission and believe that I have an appropriate level of expertise to confirm that it is of an acceptable scientific standard, however I have significant reservations, as outlined above.

# Response

**Published:** 2021-12-07

**Authors:** kinmeng cheng

**Affiliations:** Multimedia University, Malaysia

1. I have strengthened the abstract, introduction and included gamified learning to guide the readers on domain of study.
2. Literature has been enriched with gamified learning and recycling.
3. Methods has been strengthened with a clearer explanation of the EGL activity and a new Figure 2 to show the flow of the data collection activity.
4. Results and discussion has improved accordingly and social influence has been addressed, deeper meaning based on the results.
5. Implications have shown that the research from table 1 can be a potential reference for future expansion of the study for quantitative research. The four themes have been further discussed in the conclusion and implication section.

# Peer review: Reviewer response for version 1

**DOI:** 10.5256/f1000research.76364.r93524

**Published:** 2021-09-09

**Authors:** Herdiyan Maulana

**Affiliations:** 1 Faculty of Psychology, Universtias Negeri Jakarta, Jakarta Selatan, Indonesia

I appreciate the authors for bringing a very interesting yet important research theme into the table. The paper presented an insightful message about the potential uses of gamified based intervention to enhance youth recycling intention. However, there are number of concerns about the way in which the paper is presented. The following suggestions are intended to assist the authors in improving the quality of their paper.

1. The introduction need to be refined, there are numbers of unclear and inefficient sentences:
  1) " *Most countries have long ago adopted recycling to preserve the environment’s health." *This sentence is ambiguous, what do the authors mean by the environment's health? This sentences also need for further grammar check.
  2) The sentence in paragraph 2 " *A study conducted in 2017 and 2018 shows a negative attitude towards recycling in many countries, claiming that recycling education and infrastructure are insufficient". *The authors indicated "A study" but the citation refers to two different studies. This sentence also not clear, what do the authors mean by "a negative attitude?" please clarifiy.
2. Table 1. needs to be clarified. It may an idea to add study type, findings, and recommendations column for cited papers.
3. I also notice that some papers included in Table 1. did not clearly state the effectiveness of gamified method as a potential way to enhance people's recycling intention. e.g. Study by Betaubun & Nasrawati (2020), which according to the author, can be used as a reference to show the use of gamified approach for enhance recycling intention, was as a cross-sectional study. To provide strong evidences for the potential used of gamification method to enhance recycling intention, I suggest the authors to cite an experiment based publications.
4. As this is a qualitative study, a research question addressing the effectiveness of the program might be problematic. Objective indicators and systematic measures should be applied to answer if the program is really effective. Rather than testing the effectiveness, this study may focus on describing subject's subjective experiences using the program, and how their experiences benefit for developing the program. Further, the author may describe in more detail about the game itself. Why and how the game is delivered, e.g., duration, elements, self-guided or full-guided, online/offline, and system requirements of the program.
5. Braun & Clarke (2006) thematic analysis comprises series of steps when analyzing the qualitative transcriptions. The steps including how the authors develop low-order and high order themes. Please consider to explain the analysis steps in more detail. 
6. The authors may need to explain in a bit more detail about the conceptual model (figure 2). How did the authors come up with this model? What thematic analysis results lead to this model?
7. Suggestion to test the model using quantitative method should be added.

Is the work clearly and accurately presented and does it cite the current literature?

Partly

If applicable, is the statistical analysis and its interpretation appropriate?

Not applicable

Are all the source data underlying the results available to ensure full reproducibility?

Yes

Is the study design appropriate and is the work technically sound?

Partly

Are the conclusions drawn adequately supported by the results?

Partly

Are sufficient details of methods and analysis provided to allow replication by others?

Partly

Reviewer Expertise:

Thematic analysis study, Well-being, Online mental health services, well-being, social psychology

I confirm that I have read this submission and believe that I have an appropriate level of expertise to confirm that it is of an acceptable scientific standard, however I have significant reservations, as outlined above.

## References

[1] : English for Specific Purpose: Revitalizing Climate Change Awareness Using Digital Literacy and Gamification for Engineering Faculty in Papua. *Jurnal Iqra' : Kajian Ilmu Pendidikan* .2020;5(2) : 10.25217/ji.v5i2.1181 68-82 10.25217/ji.v5i2.1181

[2] : Using thematic analysis in psychology. *Qualitative Research in Psychology* .2006;3(2) :77-101

# Response

**Published:** 2021-12-07

**Authors:** kinmeng cheng

**Affiliations:** Multimedia University, Malaysia

Thank you so much for the review. I have made the changes accordingly based on the comments.

 1) Unclear statements regarding to environment's health and sentence coherency have been changed and clarified.

 2 and 3) Table has two new added columns on study type and recommendations. I have removed the study by Betaubun & Nasrawati (2020) and replaced it with a new empirical article "Advocating recycling and encouraging environmentally friendly habits through gamification: An empirical investigation" which provides stronger evidences for the effectiveness of gamification in recycling intention.

 4) I have enriched the conclusion and discussion part to show how the EGL effectively improves youths' motivation to recycle. Social influence is emphasised in several sections of the paper.

 5) 6 phases of thematic analysis have been added in 3.1 and leading each of the steps in the following sections to provide a clearer reading for the readers.

 6) Additional explanation and improvement on the conceptual framework part.

 7) Suggestions for future quantitative study have been added.
